# Machine learning model for predicting acute kidney injury progression in critically ill patients

**DOI:** 10.1186/s12911-021-01740-2

**Published:** 2022-01-19

**Authors:** Canzheng Wei, Lifan Zhang, Yunxia Feng, Aijia Ma, Yan Kang

**Affiliations:** 1 Department of Critical Care Medicine, West China Hospital of Sichuan University, Chengdu, 610041 China; 2 Department of Gastroenterology, West China Hospital of Sichuan University, Chengdu, 610041 China; 3 Department of Nephrology, Mianyan Central Hospital, University of Electronic Science and Technology of China, Chengdu, 621000 China

**Keywords:** Acute kidney injury, Critical care, Logistic Models, Extreme gradient boosting

## Abstract

**Background:**

Acute kidney injury (AKI) is a serve and harmful syndrome in the intensive care unit. Comparing to the patients with AKI stage 1/2, the patients with AKI stage 3 have higher in-hospital mortality and risk of progression to chronic kidney disease. The purpose of this study is to develop a prediction model that predict whether patients with AKI stage 1/2 will progress to AKI stage 3.

**Methods:**

Patients with AKI stage 1/2, when they were first diagnosed with AKI in the Medical Information Mart for Intensive Care, were included. We used the Logistic regression and machine learning extreme gradient boosting (XGBoost) to build two models which can predict patients who will progress to AKI stage 3. Established models were evaluated by cross-validation, receiver operating characteristic curve, and precision–recall curves.

**Results:**

We included 25,711 patients, of whom 2130 (8.3%) progressed to AKI stage 3. Creatinine, multiple organ failure syndromes were the most important in AKI progression prediction. The XGBoost model has a better performance than the Logistic regression model on predicting AKI stage 3 progression. Thus, we build a software based on our data which can predict AKI progression in real time.

**Conclusions:**

The XGboost model can better identify patients with AKI progression than Logistic regression model. Machine learning techniques may improve predictive modeling in medical research.

## Introduction

Acute kidney injury (AKI) is a common syndrome in intensive care unit with an incidence of nearly 50% [1]. It is characterized by sudden increase of serum creatinine and decrease of urine volume [2]. The survival rate of patients with AKI will decrease, which may relate to the duration of AKI [1]. A previous study found that comparing to patients with AKI duration of less than 7 days, the 1-year survival of patients with AKI lasting the entire hospital stay decreased from 90% to 44% [3]. According to the KDIGO criteria, AKI is classified into stage 1, stage 2, and stage 3 for severity [2]. Comparing to the patients with AKI stage 1/2, the patients with AKI stage 3 have higher in-hospital mortality [1] and risk of progression to chronic kidney disease (CKD) [4]. Therefore, early prediction of progression of AKI stage 1/2 to AKI stage 3 is of great importance. It is an alert for clinicians to prompt measures to avoid additional kidney damage or delay in recovery [5].

Currently, few methods were developed to predict AKI stage 1/2 to AKI stage 3. Furosemide stress test (FST) was considered as a robust predictive approach to identify who will progress to AKI stage 3 [6]. However, the clinical application has been hampered for several reasons such as lacking of high quality RCT [5], not stable for patients with unstable hemodynamics [7], no standardization of dosage and time [8] and ambiguous effect of other factors such as fluid balance and diuretic on the outcome [9].

Machine learning is a series of algorithms with set objective and without being explicitly programmed. It performs well in development of prediction model and has been widely used in medical data in recent years [10]. Machine learning technology may be helpful to establish a robust prediction model predicting AKI stage 1/2 to AKI stage 3. Currently, there are studies predicting AKI by machine learning [11–13]. However, there is no study in predicting AKI progression by machine learning. In this study, we developed prediction models to predict AKI stage 3 progression by using machine learning techniques (extreme gradient boosting) and Logistic regression.

## Method

### Data source

MIMIC-III (Medical Information Mart for Intensive Care III) is a large, de-identified comprehensive data set. It includes patients from the ICU at Beth Israel Deaconess Medical Center in Boston, Massachusetts from 2001 to 2012 [14]. This database includes general information, vital sign measurements, laboratory test results, and so on. As this study was an analysis of a third-party anonymous public database that has been approved by the Institutional Review Board (IRB), IRB approval from our institution was waived.

### Participants

Definition of AKI was: an increase in serum creatinine of 0.3 mg/dl or 50% from the baseline value or urine output < 0.5 ml/kg h [2]. This definition was consistent with the recommendations given by the Kidney Disease Improving Global Outcomes (KDIGO) criteria. The critically ill patients were included if their primary diagnosis was AKI stage 1/2. Patients who are younger than 18 years old or suffering from chronic kidney disease (CKD) were excluded. Furthermore, Patients who received RRT or progressed to AKI stage 3 within 72 h or over 28 days of first AKI diagnosis were also excluded.

### Predictors of model

We collected clinical and laboratory variables obtained within 72 h before and after the AKI diagnosis. For some variables measured multiple times in these 6 days, the outcome closest to the date of diagnosed AKI will be included in the model. We analyzed age and vital signs including heart rate, blood pressure, respiratory rate, and temperature. Besides, we followed the factors of other studies including sodium, potassium, glucose, creatinine, lactate, blood urea nitrogen (BUN), anion gap, PaO2, and pH [15]. Furthermore, we also analyzed participants whether received vasoactive drugs, cardiac surgery, mechanical ventilation, and whether have sepsis, respiratory failure, and multiple organ failure syndromes (MODS) [16]. Specifically, creatinine was calculated the mean of measurement within 6 days because serial measurements have better predictive capability than single time-point [17]. As for FST, we calculate the patient’s mean hourly urine output volume over 6 hours after receiving furosemide [18].

### Data preprocessing

Variables with missing values of more than 70% were excluded because of possible bias from missing data. Extreme gradient boosting (XGBoost) can automatically process missing values. As for the Logistic regression model, we complete missing values using the multiple imputation method in scikit-learn [19]. In this algorithm, we models that a feature column is designated as output and other feature columns are treated as inputs by using that estimating for imputation iteratively [20]. And, most classification algorithms will only perform optimally when the number of samples of each class is roughly the same [21]. The low rate of progression patients (8.3%) may have bad effect on model generalization. A combination of over-sampling and under-sampling [22] can balance the proportion of patients in the two groups. This algorithm first over-sample minority class examples by generating examples and then under-sample majority class examples by deleting examples [23]. We divided the original data into a train set (70%) and a teat set (30%). Both XGBooost and Logistic regression were train on the train set and assessed on the test set.

### Model selection and development

We compared characteristics between groups by Student t-test. In addition, as for categorical and nonnormal variables, we used the Chi-square test and the Kruskal–Wallis Rank Sum Test respectively.

Logistic regression model to predict AKI progression was established by forward selection and backward elimination. In this process, we iteratively assess model by Akaike Information Criteria (AIC) after including or excluding a feature. AIC give consideration to the features incorporated into the model and the predictive performance [23]. Therefore, the final model have best prediction performance and contain the fewest features.

Extreme gradient boosting (XGBoost) is an ensemble method of machine learning based on decision trees [24]. The decision trees was set as the weak learners and binary logistic was set as objective. We iteratively re-fit the weak classifier (decision tree) to the residuals of the previous model. Each iteration adds a tree to the existing tree to fit the residuals between the predicted and true values of the previous tree. XGBoost hyperparameters included learning rate, maximum depth of trees, minimum child weight, subsample ratio, minimum split loss, parameters for subsampling of columns and parameters for regularization. In this study, we performed 100 iterations of the cross-validation process, which is also the default and recommended value [25]. All analyses were performed using Python, version 3.7.9.

In train set, the data was randomly divided into five equal-sized subsamples. Four subsamples were used to train the model and then validated in the remaining one. On this basis, hyperparameters were tuned for the higher area under receiver operating characteristic curve (AU-ROC) which can evaluate the predictive ability of the model. We used grid search which can cycle through tuning and scoring to select the hyperparameters. Learning curve that demonstrates AU-ROC of the model by changing the subsample ratio helps prevent overfitting or overfitting. After choosing the hyperparameters, the XGBoost was trained for the final model on the whole train set. Then, the model was estimated on the test set.

## Results

### Participants

**Fig. 1:**
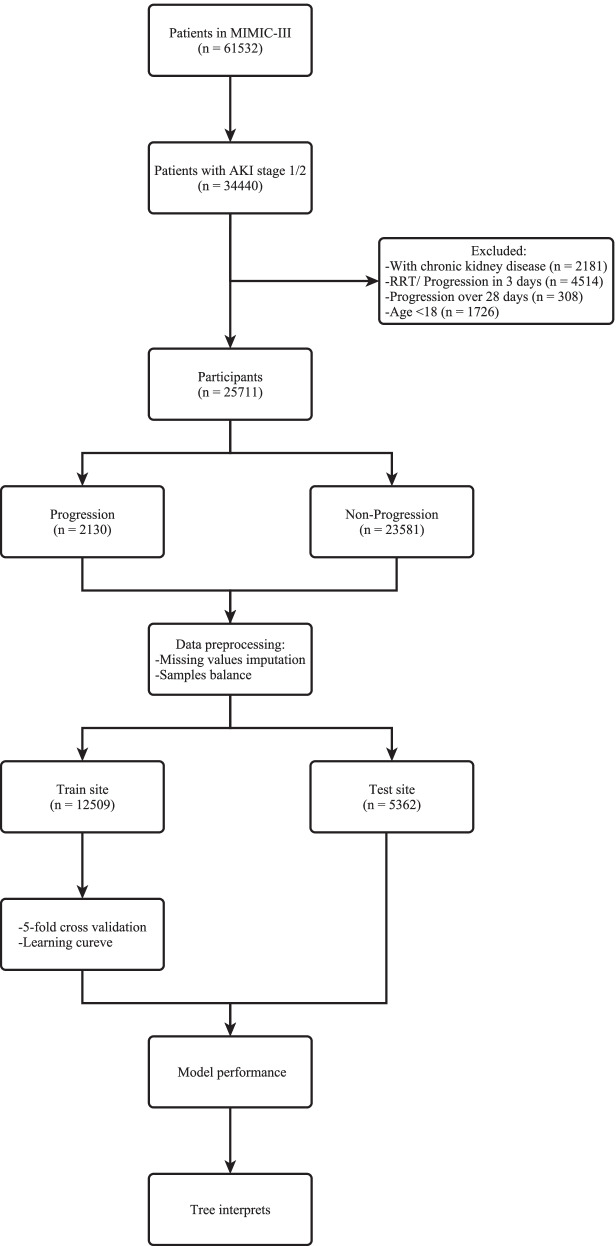
Flow chart of patient selection and data processing

Of the 61,532 patients in MIMIC-III, 34,440 (56.0%) patients were diagnosed AKI stage 1/2 of the first AKI diagnosis. 8729 patients were excluded according to the pre-designed criteria. A total of 25,711 patients were included in our analysis; 2130(8.3%) patients finally progress to AKI stage 3, and 23,581 (91.7%) patients did not (Fig. 1).

Characteristic differences between groups are shown in Table 1. There were more women (44.5% vs. 41.3%; *p* = 0.005) and emergency patients in progression group. Fewer patients underwent cardiac surgery in the progression group than in the non-progression group (72.9% vs. 80.8%; *p* < 0.001). The creatinine (2.4 ± 2.0 vs. 1.1 ± 0.9 mg/dl; *p* < 0.001) and the BUN (39.0 ± 26.2 vs. 23.5 ± 17.0 mg/dl; *p* < 0.001) were higher, and FST (93.6 ± 108.6 vs. 108.8 ± 113.1 ml/h; *p* < 0.001) was lower in the progression group. The progression group had higher heart rate (90.2 ± 21.2 vs. 86.1 ± 17.9; *p* < 0.001), higher respiratory rate (20.3 ± 6.9 vs. 19.1 ± 5.9; *p* < 0.001) than the non-progression group. As for laboratory data, lactate (2.5 ± 2.3 vs. 2.1 ± 1.6 mmol/l; *p* < 0.001), glucose (144.9 ± 105.2 vs. 136.3 ± 68.4 mg/dl; *p* < 0.001), and potassium (4.3 ± 0.8 vs. 4.2 ± 0.6 mmol/l; *p* < 0.001) were higher in progression group. But sodium (138.5 ± 4.6 vs. 137.6 ± 5.6 mmol/l; *p* < 0.001) was lower in progression group. And progression group had higher rate of mechanical ventilation (34.3% vs. 28.2%; *p* < 0.001), higher rate of MODS (94.1% vs. 59.2%; *p* < 0.001), higher rate of respiratory failure (63.7% vs. 34.0%; *p* < 0.001), higher rate of sepsis (41.4% vs. 14.7%; *p* < 0.001).

**Table 1:** Characteristics between progression and non-progression groups

Variables	Overall	Non-progression (n = 23,581)	Progression (n = 2130)	*P* value
Gender, n (%)				0.005
Male	15,018 (58.4)	13,836 (58.7)	1182 (55.5)	
Female	10,693 (41.6)	9745 (41.3)	948 (44.5)	0.005
Age, median [Q1,Q3]	66.0 [55.0,77.0]	66.0 [55.0,77.0]	65.5 [53.0,76.0]	0.001
Ethnicity, n (%)				< 0.001
Asian	503 (2.0)	470 (2.0)	33 (1.5)	
Black	2042 (7.9)	1805 (7.7)	237 (11.1)	
Hispanic	718 (2.8)	649 (2.8)	69 (3.2)	
Native	12 (0.0)	10 (0.0)	2 (0.1)	
Other	656 (2.6)	602 (2.6)	54 (2.5)	
Unknown	3106 (12.1)	2835 (12.0)	271 (12.7)	
White	18,674 (72.6)	17,210 (73.0)	1464 (68.7)	
Admissions_type, n (%)				
ELECTIVE	4511 (17.5)	4342 (18.4)	169 (7.9)	< 0.001
EMERGENCY	20,450 (79.5)	18,559 (78.7)	1891 (88.8)	
URGENT	750 (2.9)	680 (2.9)	70 (3.3)	
ICU_type, n (%)				< 0.001
CCU	3647 (14.2)	3288 (13.9)	359 (16.9)	
CSRU	6500 (25.3)	6253 (26.5)	247 (11.6)	
MICU	8679 (33.8)	7681 (32.6)	998 (46.9)	
SICU	3937 (15.3)	3561 (15.1)	376 (17.7)	
TSICU	2948 (11.5)	2798 (11.9)	150 (7.0)	
Cardiac surgery, n (%)	20,596 (80.1)	19,043 (80.8)	1553 (72.9)	< 0.001
Respiratory failure, n (%)	9381 (36.5)	8025 (34.0)	1356 (63.7)	< 0.001
Mechanical ventilation, n (%)	7380 (28.7)	6650 (28.2)	730 (34.3)	< 0.001
MODS, n (%)	15,961 (62.1)	13,958 (59.2)	2003 (94.0)	< 0.001
Spesis, n (%)	4358 (16.9)	3476 (14.7)	882 (41.4)	< 0.001
Vasoactive_drugs, n (%)	8098 (31.5)	7425 (31.5)	673 (31.6)	0.937
Vital signs, mean (SD)				
Blood pressure (mmHg)	76.6 (16.4)	76.7 (16.0)	76.3 (20.3)	0.527
Heart rate	86.4 (18.2)	86.1 (17.9)	90.2 (21.2)	< 0.001
Respiratory rate	19.2 (6.0)	19.1 (5.9)	20.3 (6.9)	< 0.001
Temperature ($^\circ \mathrm{C}$)	36.9 (0.6)	36.9 (0.6)	36.8 (0.7)	< 0.001
Laboratory variables, mean (SD)				
Anion gap (mEq/l)	13.5 (3.6)	13.3 (3.5)	15.4 (4.5)	< 0.001
PaO2 (mmHg)	128.0 (77.8)	128.5 (77.7)	123.5 (79.7)	0.016
pH	7.4 (0.1)	7.4 (0.1)	7.4 (0.1)	< 0.001
Glucose (mg/dl)	137.0 (72.2)	136.3 (68.4)	144.9 (105.2)	< 0.001
Lactate (mmol/l)	2.1 (1.7)	2.1 (1.6)	2.5 (2.3)	< 0.001
Sodium (mmol/l)	138.4 (4.7)	138.5 (4.6)	137.6 (5.6)	< 0.001
Potassium (mmol/l)	4.2 (0.6)	4.2 (0.6)	4.3 (0.8)	< 0.001
BUN (mg/dl)	24.8 (18.5)	23.5 (17.0)	39.0 (26.2)	< 0.001
Creatinine (mg/dl)	1.2 (1.1)	1.1 (0.9)	2.4 (2.0)	< 0.001
FST (ml/h)	107.8 (112.9)	108.8 (113.1)	93.6 (108.6)	< 0.001

### The logistic regression model

The results of the Logistic regression model are shown in Table 2 and Additional file 2: Fig. S1. After excluding the variables with high colinearity through the variance inflation factor (VIF) [26], the final variables included in the analysis are as follows. As expected, with MODS (odds ratio [OR] 1.55; 95% confidence interval 1.50 to 1.60), sepsis (OR 1.71; 95% CI 1.60 to 1.82), respiratory failure (OR 1.47; 95% CI 1.41 to 1.54), and creatinine (OR 1.20; 95% CI 1.15 to 1.25) were associated with increased probability of AKI progression(Table 1). Besides, BUN, lactate, and so on are also considered to be associated with AKI progression. On the contrary, male (OR 0.91; 95% CI 0.87 to 0.95) and previous cardiac surgery (OR 0.86; 95% CI 0.81 to 0.91) were associated with a reduced likelihood of AKI progression (Table 2).

**Table 2:** Logistic regression model with stepwise variable selection

Variables	OR	95% CI	*P* value
Gender	0.91	0.87, 0.95	< 0.001
Admissions type	1.16	1.1, 1.22	< 0.001
Cardiac surgery	0.86	0.81, 0.91	< 0.001
Respiratory failure	1.47	1.41, 1.54	< 0.001
Mechanical ventilation	1.00	0.96, 1.05	0.880
MODS	1.55	1.50, 1.60	< 0.001
Spesis	1.71	1.6, 1.82	< 0.001
Vasoactive drugs	1.04	0.99, 1.09	0.101
BUN	1.01	1.01, 1.01	< 0.001
Creatinine	1.20	1.15, 1.25	< 0.001
PaO2	1.00	1.00, 1.00	0.0535
Glucose	1.00	1.00, 1.00	0.187
Lactate	1.03	1.02, 1.05	< 0.001
FST	1.00	1.00, 1.00	< 0.001

### The XGBoost model

**Fig. 2:**
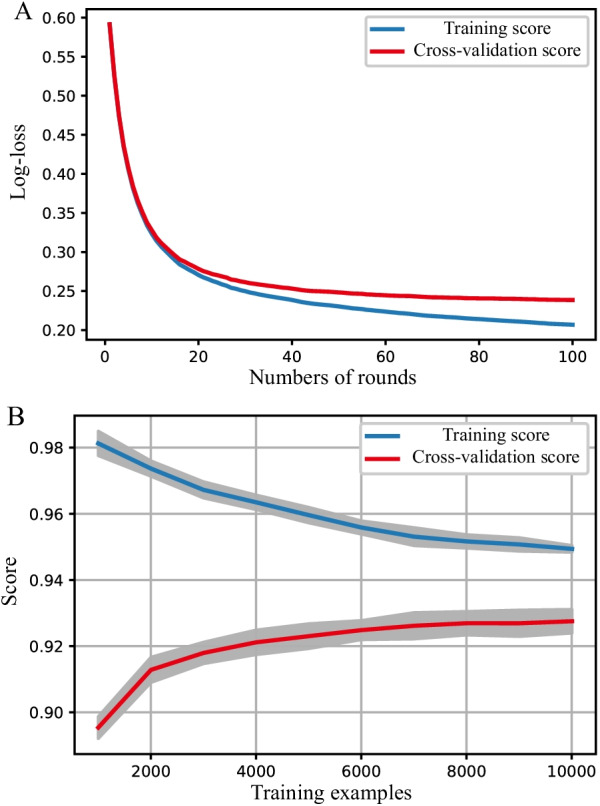
The training process of the extreme gradient boosting machine. **A** Cross-validation during XGBoost hyperparameter tuning. The log-loss value for the training and testing datasets is shown in the vertical axis. The dashed vertical line indicates the number of rounds with the minimum log-loss in the test sample. **B** Learning curve of the XGBoost model after hyperparameter tuning. AU-ROC value for the testing and training datasets is shown in the vertical axis. With the subsample ratio increasing, AU-ROC of training datasets decreases, and AU-ROC of testing datasets increases. The training score is always higher than the test score

Determining by grid search, the hyperparameters used in our analysis were set as learning rate = 0.19, minimum child weight = 8, maximum tree depth = 3, and the number of rounds = 100 (Additional file 1: Table S1). With these hyperparameters, the training score increases as the number of rounds increases, and the cross-validation score test log-loss is only slightly higher than the training log-loss as the tree grows (Fig. 2A).

Learning curve demonstrated the cross-validation on train set and represent the generalization performance of the model as a function of the size of the training set [27]. Model was train on four-fifths of train set and validate on one-fifth of train set iteratively by AU-ROC. As the size grows, the difference between performance of the model on the train and test sets gradually narrowed (Fig. 2B) suggesting the model is generalizable and robust [28].

### Model performance

**Fig. 3:**
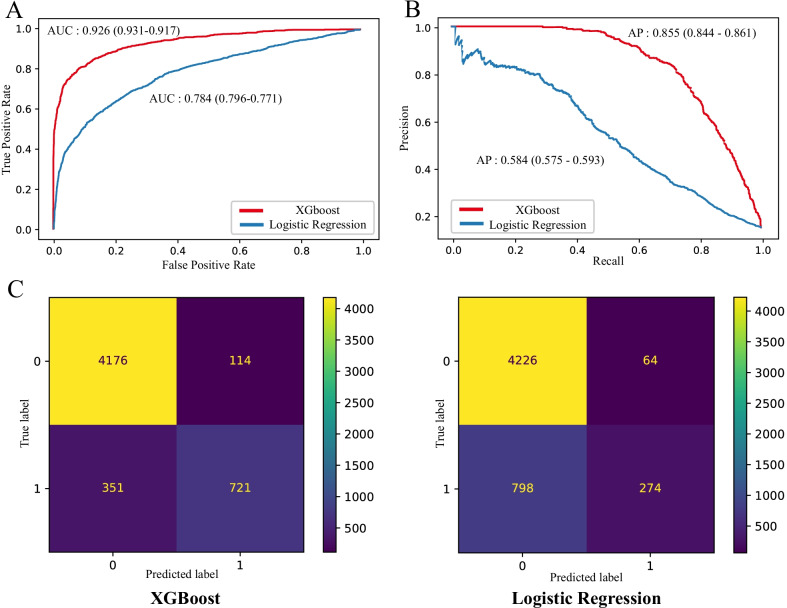
Performance of the XGBoost and Logistic regression model **A** Receiver operating characteristic curve for estimating the discrimination between the Logistic regression model and the XGBoost model. **B** Precision–recall curve for estimating the discrimination between the Logistic regression model and the XGBoost model. **C** Confusion matrix of the Logistic regression model and XGBoost model. The color represents the number of patients. Whether progress is represented by numbers

The model was evaluated using receiver operating characteristic curve (ROC) and precision–recall curve (PRC) on test set. AU-ROC of XGBoost is significantly higher than the Logistic regression model (AU-ROC 0.926; 95% CI 0.917 to 0.931 vs. 0.784; 95% CI 0.771 to 0.796, respectively; Fig. 3A). And area under precision–recall curve (AU-PRC) of XGBoost is also significantly higher than the Logistic regression model (AU-PRC 0.855; 95% CI 0.844 to 0.861 vs. 0.584; 95% CI 0.575 to 0.593, respectively; Fig. 3B) We also showed the confusion matrix for the two models in predicting AKI progression (Fig. 3C).

### Tree interprets

SHAP (SHapley Additive exPlanations) is a game theory method which can intuitively and accurately explain the output of machine learning model [29]. As for this dichotomous classifier, the higher SHAP value, the higher probability of AKI progression. The base value is defined as the output when each variable in the training dataset is averaged, which can represent the average of the sample. With original data, we calculate the base value is − 0.468. Therefore, the average of these patients is unlikely to progress to AKI stage 3, which can be explained by the relatively low proportion of progression patients (8.3%).

**Fig. 4:**
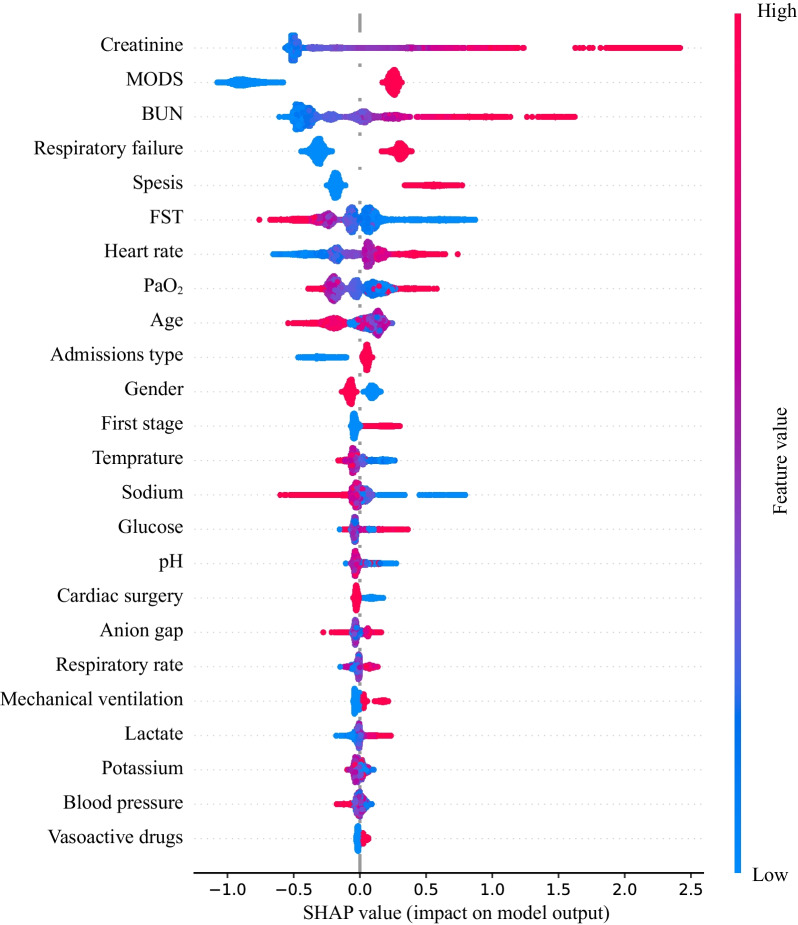
SHAP value of XGBoost model output. SHAP value of all patient output. Each point represents a variable for an observation. The color of the point is determined by its relative height in the variable. The blue represents lower and the red represents higher

SHAP value can intuitively show features each contribution to push the model output from the base value (Additional file 3: Fig. S2). SHAP value can be considered as a quantified contribution. We can easily find the contribution of all features and which contribution is most (Fig. 4). The features are ordered in order of importance. Feature importance was calculated by the mean contribution of every observation, which is equal to the traditional method [30]. The serum creatinine was the most important variable, followed by MODS and respiratory failure. The specific importance of each variable is shown in Additional file 4: Fig. S3.

### Software for prediction

**Fig. 5:**
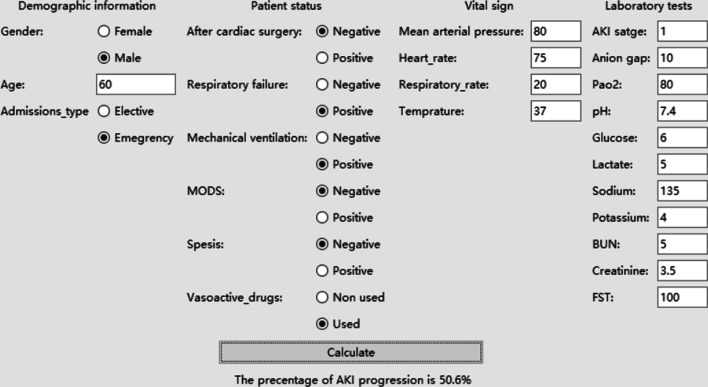
The calculator for predcting AKI progression in real time. Feature importance was calculated by the mean contribution of every observation, which is equal to the traditional method. Abbreviations and annotations: BUN, blood urea nitrogen; FST, furosemide stress test; MODS, multiple organ failure syndromes

A web calculator based on this data was developed for clinicians to predict patients’ AKI progression (https://260147169.github.io/AKI-progression/AKI-progression-calculator.html) (Fig. 5). After inputting the corresponding data of the patient, the prediction can be made automatically. Besides, misssing value is acceptable, because XGBoost can complete automatically.

## Discussion

In this study, we analyzed data in MIMIC-III and proposed machine learning models predicting AKI progression. The machine learning model had excellent performance in predicting AKI stage 1/2 to AKI stage 3 with ROC of 0.926, which was significantly better than the performance of logistics model (0.926 [95% CI 0.917–0.931] vs. 0.784 [95% CI 0.771–0.796]). By interpreting the importance of each variable of the model (Additional file 4: Fig. S3), we found creatinine and MODS were more important than others.

Comparing AKI stage 1, patients with AKI stage 3 have higher risk of mortality in the intensive care unit (odds ratio (OR) 2.19 [95% CI 1.44–3.35] vs. OR 7.18 [95% CI 5.13–10.04]) [31]. Therefore, predicting AKI progression is always one of research highlights. FST, a method for predicting AKI progression, had desirable prediction ability, which of AU-ROC was 0.88 [6]. Our model and previous FST studies have comparable prediction ability (AU-ROC 0.926 vs. 0.88). However, FST has some limitations including not stable for patients with unstable hemodynamics [7] and no standardization of dosage and time [8]. Our model is based on vital signs and laboratory data, which are easily assessable in most institutions. These features are also significant predictors of AKI in the others model such as sepsis and creatinine [11, 13]. Real-time automated prediction and analysis of main cause are also advantages of our study.

**Fig. 6:**
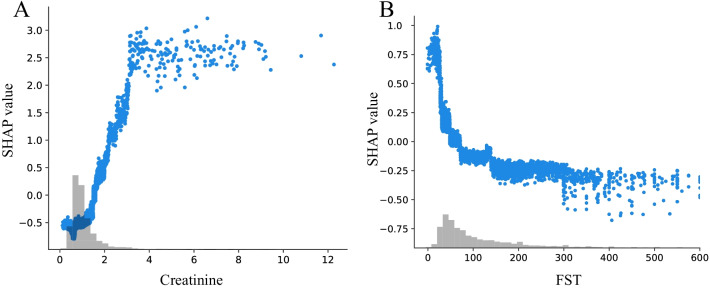
SHAP value for single variable. **A** SHAP value for creatinine. SHAP value increases with the increase of creatinine until creatinine probably reaches 5 mg/dL. **B** SHAP value for FST. SHAP value decreases with the increase of FST until FST reach 100 ml/h

We employed visualization function in SHAP [32] to find the effect of the specific value of each variable on model output. There are some factors contributing most including creatinine, MODS, BUN, sepsis and so on. The KDIGO criteria proposed some similar exposures that may cause AKI including sepsis and shock [2]. Advanced age, underlying CKD, sepsis, and cardiac surgery were also proposed as risk factors for AKI [1, 33]. SHAP value was found to increase with the increase of creatinine until creatinine probably reach 3 mg/dl (265.2 $\upmu$mol/L) (Fig. 6A). This is in line with the mainstream view in clinicians [34, 35]. The relationship between SHAP value and FST (Fig. 6B) is consistent with previous studies that FST < 100 ml/h increases the risk of AKI progression [7, 18].

Our study found that machine learning has better performance than logistic regression, which is similar to the previous prediction study of AKI [13, 36]. Lee et al gradient boosting tree has better performance than logistic regression (0.78 vs. 0.69) on predicting AKI after cardiac surgery [13]. Zhang et al found the machine learning model outperformed the logistic regression model (AU-ROC 0.860 vs. 0.728) in differentiating between the volume-responsive and volume-unresponsive AKI [36]. The advantages of machine learning include ability of capturing complex non-linear relationships [37] and focusing more on misclassified observations , especially when the sample size is large enough [37]. And, machine learning can automatically input missing values and give the prediction as soon as possible for intervention in time.

A limitation of this study is a retrospective study with inevitable bias. And, the proportion of patients who eventually progressed to AKI stage 3 (8.3%) is significantly lower than that without progression (91.7%). Even if we use the algorithm to balance the sample, it may still have bad effect on model generalization and reliability. Furthermore, external validation is still required in the following study.

## Conclusion

We collected data from MIMIC-III and proposed a predicting model for AKI progression from stage1 to stage 2/3 by machine learning. The model had excellent performance in predicting AKI progression and was significantly better than the performance of logistics model. In the final model, creatinine, MODS and BUN were factors contributing most. The reasons of performance gap and important factors require further study.

## Supplementary information

**Additional file 1: Table S1.** Hyperparameters of the XGboost model

**Additional file 2: Figure S1.** Results of the Logistic regression model. The odds ratio[OR] of each feature is shown in the horizontal axis

**Additional file 3: Figure S2.** The waterfall plot of a single patient. The SHAP value each feature is shown in the horizontal axis.

**Additional file 4: Figure S3.** Feature importance derived from the XGBoost model. The importance of each feature calculated by the average of the absolute value of SHAP value is shown in the horizontal axis

## Data Availability

The datasets used for the analysis in the current study are available from the corresponding author on reasonable request.
