# Supplementary material for: Machine learning model for predicting acute kidney injury progression in critically ill patients
Source: BMC Med Inform Decis Mak. 2022 Jan 19;22:17. doi: 10.1186/s12911-021-01740-2 (PMC8772216; doi:10.1186/s12911-021-01740-2)
Supplement: Supplementary file 1 — Additional file 1: Table S1. Hyperparameters of the XGboost model [file 12911_2021_1740_MOESM1_ESM.docx]

**Supplementary Table 1. Hyperparameters of the XGboost model**

| **Hyperparameters** | **Value** |
| --- | --- |
| booster | gbtree |
| colsample_bynode | 0.8 |
| colsample_bytree | 0.7 |
| colsample_bylevel | 0.7 |
| gamma | 0 |
| learning_rate | 0.19 |
| max_delta_step | 0 |
| max_depth | 3 |
| min_child_weight | 8 |
| n_estimators |  |
| reg_alpha | 2 |
| reg_lambda | 1 |
| scale_pos_weight | 1 |
