# Supplementary figures and images for: Machine learning model for predicting acute kidney injury progression in critically ill patients
Source: BMC Med Inform Decis Mak. 2022 Jan 19;22:17. doi: 10.1186/s12911-021-01740-2 (PMC8772216; doi:10.1186/s12911-021-01740-2)

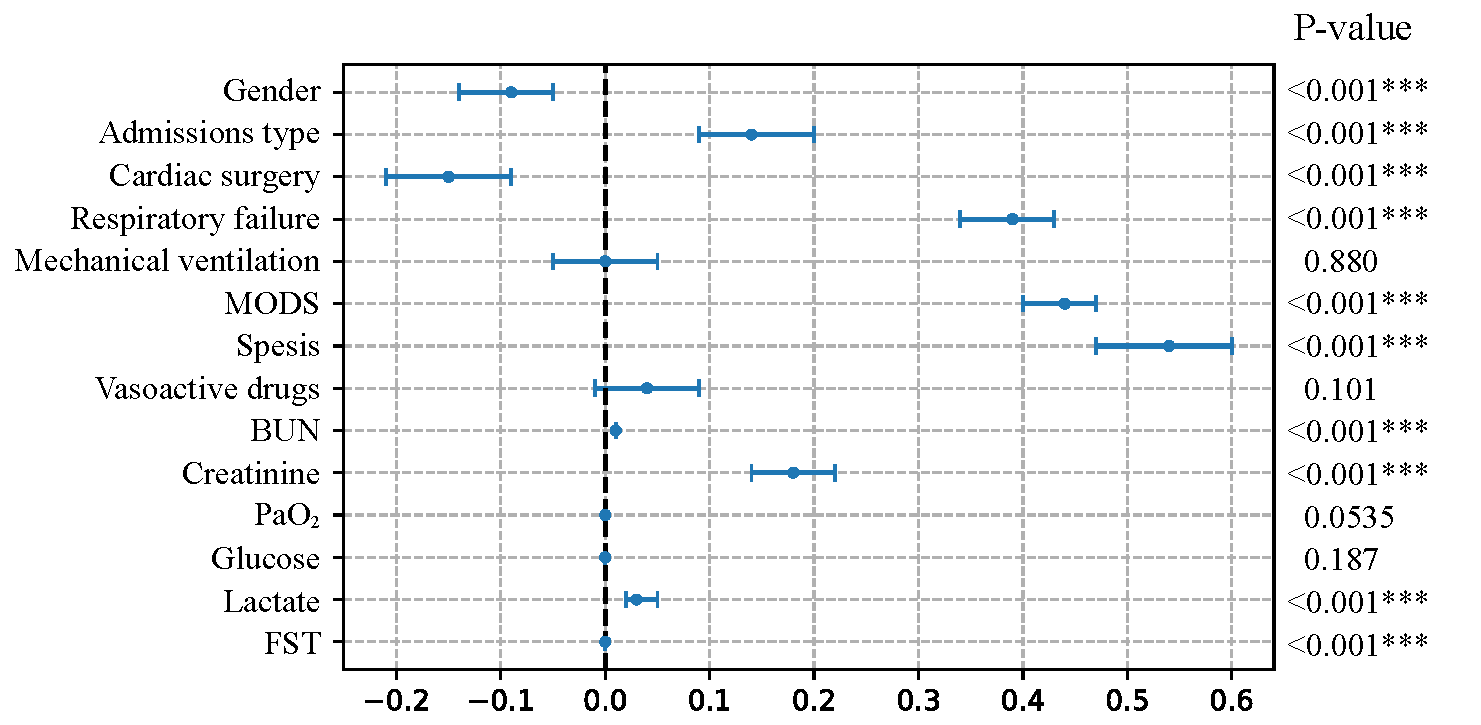
**Supplementary Figure 1. Results of the Logistic regression model**

Supplement: Supplementary file 2 — Additional file 2: Figure S1. Results of the Logistic regression model. The odds ratio[OR] of each feature is shown in the horizontal axis [file 12911_2021_1740_MOESM2_ESM.docx]

**
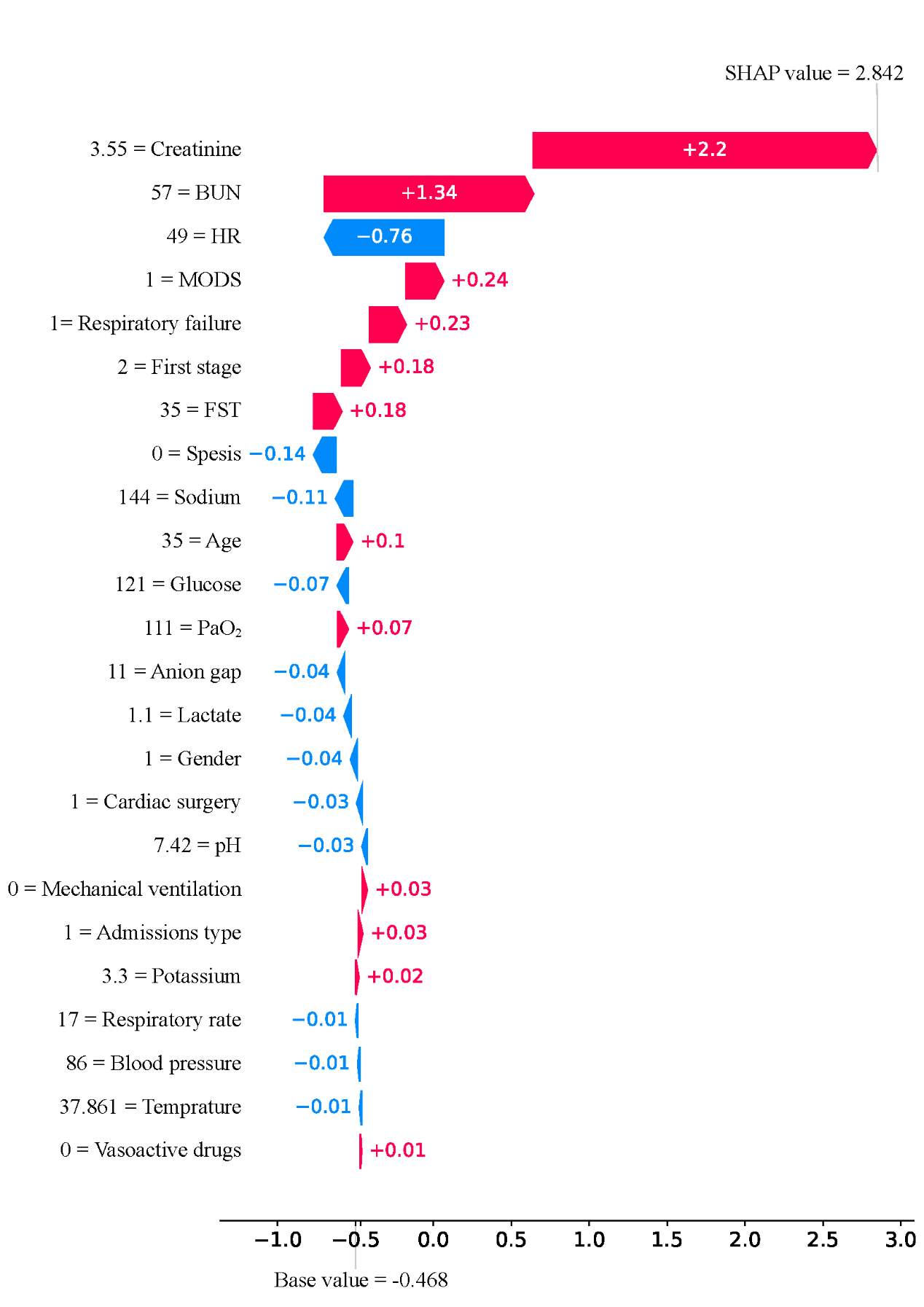
Supplementary Figure 2. The waterfall plot of a single patient**

Supplement: Supplementary file 3 — Additional file 3: Figure S2. The waterfall plot of a single patient. The SHAP value each feature is shown in the horizontal axis. [file 12911_2021_1740_MOESM3_ESM.docx]

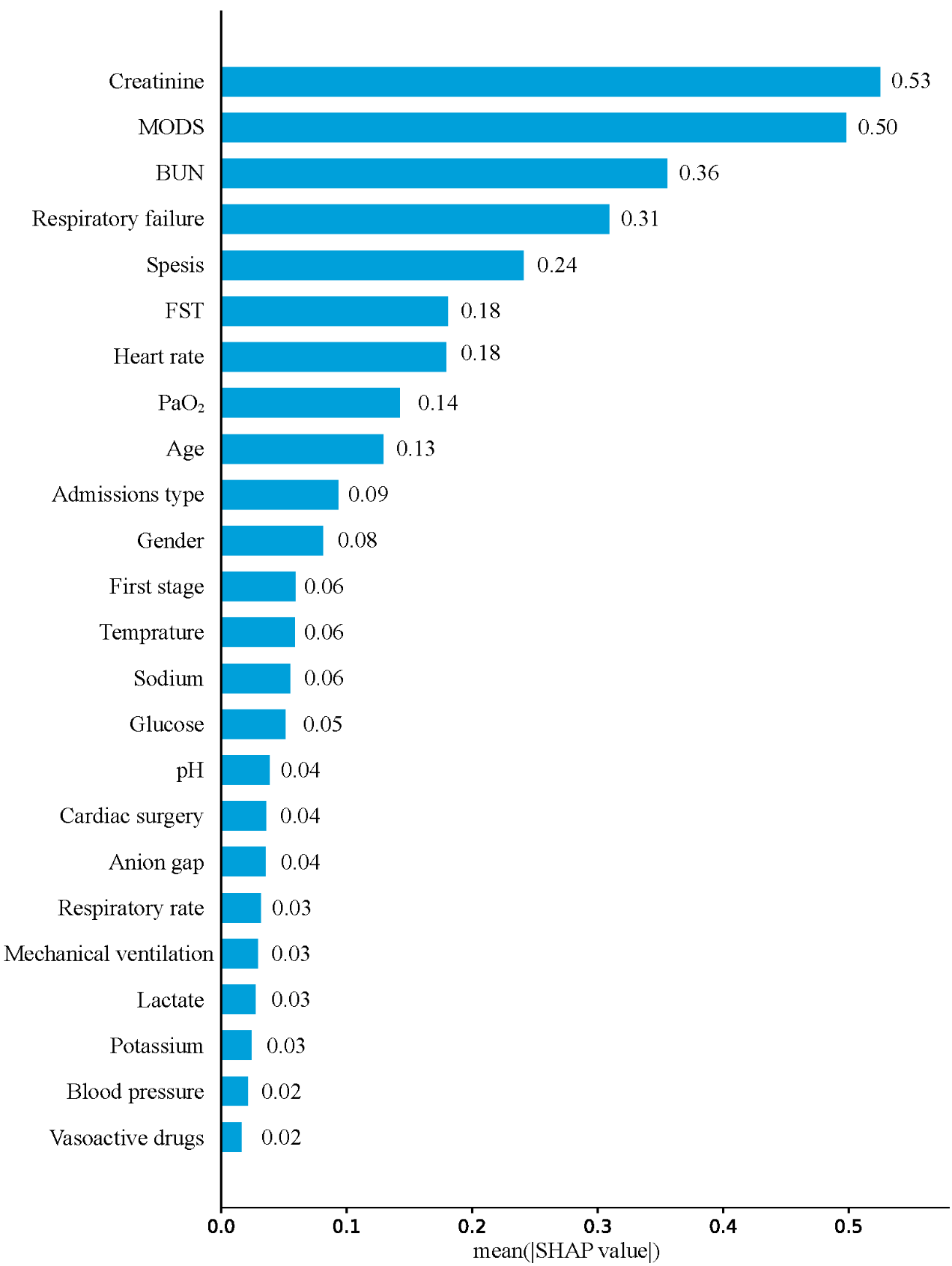
**Supplementary Figure 3.** **Feature importance derived from the XGBoost model**

Supplement: Supplementary file 4 — Additional file 4: Figure S3. Feature importance derived from the XGBoost model. The importance of each feature calculated by the average of the absolute value of SHAP value is shown in the horizontal axis [file 12911_2021_1740_MOESM4_ESM.docx]
